# Artificial intelligence: promise and peril in achieving the quadruple aim in healthcare

**DOI:** 10.3389/frai.2024.1430756

**Published:** 2024-06-19

**Authors:** William B. Weeks, Juan M. Lavista Ferres, James N. Weinstein

**Affiliations:** ^1^AI for Good Lab, Microsoft Corporation, Redmond, WA, United States; ^2^Microsoft Research, Microsoft Corporation, Redmond, WA, United States

**Keywords:** artificial intelligence, healthcare, shared decision making, social determinants of heath, population health, healthcare delivery

## Background

While there are many examples of how healthcare could create value by emulating processes used in non-healthcare industries (Gawande, 2012), healthcare is fundamentally different from other manufacturing or services industries for three main reasons: (1) healthcare prices are not transparent and are not directly paid for by the consumer; (2) unlike other industries that try to increase per-capita revenue, US healthcare aims to minimize per-capita costs by eliminating waste, improving quality, and achieving better care outcomes; and (3) healthcare cannot standardize its inputs, as patients have unique health profiles, environmental exposures, and genetic predispositions to health states (Weeks and Weinstein, 2015).

The goal of healthcare is to achieve quadruple aim: improving the patient experience, the provider experience, and healthcare quality and outcomes (equitably), while reducing per-capita healthcare expenditures (Bodenheimer and Sinsky, 2014). However, this is challenging because of the impact of social determinants of health, which account for up to 70% of patients' health outcomes (Singh et al., 2017). Further, there is substantial waste in the US healthcare system, some of which is administrative and attributable to the US third party payment system (Gaffney et al., 2023), but much of which is attributable to overuse of unnecessary products and services (Shrank et al., 2019) and excessive provision of low value care (Powers et al., 2020). As a result, the haphazard application of artificial intelligence may amplify inefficiencies and biases in the US healthcare system.

## A different approach

In the absence of health insurance reform, technological transformation of healthcare and using AI (artificial intelligence) to achieve the quadruple aim may improve healthcare efficiency in four ways (summarized in the Table 1).

**Table 1:** Areas in which artificial intelligence might promote achievement of the quadruple aim (top) and pitfalls to avoid in using artificial intelligence in that pursuit (bottom), with examples and solutions, respectively, provided.

**Areas in which artificial intelligence might promote achievement of the quadruple aim**	
**Area**	**Example**
Helping patients decide whether to obtain services	With collection of patient reported outcomes measures (PROMs), informing patient decision making by providing personalized trajectories of anticipated outcomes, given treatment options, over time.
Helping patients decide where to obtain care	With current data and PROMs, guiding patients toward providers who have the most experience and best results for patients like them.
Helping policymakers understand the relationship between social determinants of health and health outcomes	Leveraging natural experiments and other causal models to create ‘what if' scenarios that can inform policymakers' decision making.
Supporting providers' decision making	Providing real-time information on the best treatment approaches for a patient like the one in front of you.
**Pitfalls to avoid**	
**Pitfall**	**Solution**
Chasing the wrong metrics	Focus on achievement of the quadruple aim, not exclusively model performance metrics.
Not including a human in the loop	Include subject matter experts in model development, interpretation, and evaluation.
Not testing models prospectively	Test, validate, and monitor models prospectively to ensure they are meeting quadruple aim goals.
Not using responsible AI practices	Integrate responsible AI practices into model development and ensure that biases inherent in models are recognized and addressed.

### Helping patients decide whether to obtain services

Informed choice can change the dynamics of decision making and empowering well-informed consumers with objective information about treatment options, potential risks and benefits of different options, and the degree to which their choices align with their own values (Stacey et al., 2017). Decision aids are particularly helpful in preference-sensitive conditions – like lumbar spinal stenosis, where medical and surgical treatment options offer different short- and long-term risks and benefits (Asthana et al., 2024) – but can also be useful in acute care settings, as when patients are admitted to an Intensive Care Unit where issues of outcomes and futility might arise (Göcking et al., 2023). However, decision aids are under-used (The Commonwealth Fund, 2024). In the short run, for instance, AI could surface treatment options when a patient is using a search engine to seek care for a particular condition, enhance patients' knowledge of those treatment options, and empower them to ask questions of their providers and make informed choices. In the long run, should additional information be collected in the treatment and follow-up process, informed by patient reported outcomes measures collected on patients who did and did not obtain a particular procedure, AI could model outcome trajectories for patients with particular clinical and demographic constellations, helping patients identify treatment pathways consistent with their preferences and values.

### Helping patients decide where to obtain desired care

Having decided to obtain care, patients may be uninformed about where to obtain the best care for their healthcare need. There is a long history demonstrating that, for certain procedures, there is a volume-outcomes relationship wherein providers (Birkmeyer et al., 2003; Goodney et al., 2005; Sahni et al., 2016) and hospitals (Flood et al., 1984) that perform more procedures have better outcomes. Despite efforts by the Leapfrog Group (Leapfrog Hospital Survey, 2024) and the Centers for Medicare and Medicaid Services (Centers for Medicare and Medicaid Services, 2024) to provide provider- and procedure-specific information on procedural volumes, those data are not readily interpretable by patients who are making decisions about where to obtain services. Further, while provider- and hospital quality indicators abound, despite finding them useful (Marang-Van De Mheen and Vincent, 2023), patients currently rely much more on word of mouth and interpersonal skills than objective quality metrics when choosing a provider (Yahanda et al., 2016). In the short run, AI could identify high-volume and high-quality providers for particular procedures, surface additional information that patients might want to know (like gender, board certification status, medical school attended, year of graduation, and social media reviews), and aggregate those data to facilitate informed consumer choice. In the long run, emulating matching algorithms used in ecommerce websites, AI might identify the best patient-provider matches based on patients' expectations of encounters and the types of encounters and interactions providers prefer.

### Helping policymakers understand the relationship between social determinants of health and healthcare access, quality, and outcomes

The long documented relationships between social determinants of health (SDOH) and health status and outcomes are complex (Marmot et al., 1978; Blane, 1995; Chetty et al., 2016). AI can be used to inform policymakers on the magnitude, timing, and beneficiaries of returns on investment to health following effort to improve SDOH equity (Weeks et al., 2023). To date, these relationships have been associative: in the future, leveraging natural experiments and other causal models, AI might model “what if” scenarios to help policymakers prioritize SDOH interventions to maximize returns to health.

### Supporting providers' decision making

The exceedingly high volume of medical knowledge generated makes it impossible for clinicians to keep current with treatment recommendations.AI-informed clinical decision support tools have great potential use in healthcare: (Giordano et al., 2021) such tools can improve care quality, lead to more rapid diagnosis and treatment, and support equity in healthcare delivery. This support can enhance healthcare efficiency – thereby lowering per-capita costs – while improving the provider experience and patients' experiences and health outcomes, as regularly evaluated by regulatory agencies.

## Avoiding misapplication of AI in medicine

While AI has tremendous promise in helping to achieve the quadruple aim, as articulated above, we suggest four guidelines for avoiding haphazard applications of AI in medicine.

1. Avoid chasing the wrong metrics. The goal of a model is not to achieve the best area under the curve, but to have measurable positive clinical impact and to achieve the quadruple aim, using metrics defined prior to model implementation. If the model or the technology does not measurably and efficiently promote achievement of the quadruple aim, it should not be implemented.
2. Always include a human subject matter expert in the loop. Models increasingly use a ‘human in the loop' process to ensure that the model is operating as intended; in healthcare, it is critical to include a healthcare subject matter expert in the loop. Models must make sense to providers, so interpretable models and tools can allow subject matter experts to evaluate a model's utility in clinical practice, using metrics defined prior to model implementation.
3. Test, validate, and monitor models. AI models are invariably developed, tested, and refined retrospectively. While AI models have an advantage of testing results on a randomly selected held-out dataset, just as with any quality improvement effort all AI models should be prospectively tested and validated on the target population before widespread implementation, using pre-defined validation thresholds. Further, models should be monitored over time: if models are effective, they may lead to behavior change; that behavior change may change key relationships, and those changes will require new model development. Those determining whether to develop and implement AI models should consider the cumulative long-term costs of monitoring and re-developing models.
4. Use responsible AI practices Model effectiveness is intrinsically tied to the quality of data used in their training; ensuring that those data free from bias is crucial. When the data itself are not biased, subsequent decisions derived from its analysis – for instance, misapplication of models to populations that are not represented in the unbiased data - might be unfair. Particularly with health-related AI models, where stakes are significantly higher and impacts more profound, adherence to responsible AI practices is imperative.

## Conclusion

Current uses of AI applications can improve the efficiency of healthcare operations, such as scheduling, letter writing, provider in-box email responses, patient triage, and coding optimization. These uses can improve patient and provider experiences, reduce per-capita healthcare costs, and promote achievement of the quadruple aim.

However, given healthcare's many inefficiencies, the unconsidered application of AI may increase healthcare costs without advancing the quadruple aim. AI models can be expensive to develop, test, implement, and monitor: a modest increase in accuracy may not warrant the expense if the impact on patients and clinical care is not significant. An unwavering focus on and objective evaluation of how technological implementation helps achieve the quadruple aim is essential for improving healthcare efficiency and effectiveness.

## Author contributions

WW: Conceptualization, Writing – original draft. JL: Writing – review & editing. JW: Writing – review & editing.
